# Long-term Cardiovascular and All-Cause Mortality following Elective Infrarenal Repair of the Abdominal Aortic Aneurysm: A Systematic Review and Meta-analysis

**DOI:** 10.1177/15266028241304627

**Published:** 2024-12-30

**Authors:** Samira E. M. van Knippenberg, Cecilia Fenelli, Susan van Dieren, Ronak Delewi, Ron Balm, Kak Khee Yeung

**Affiliations:** 1 Department of Surgery, Amsterdam University Medical Centers, University of Amsterdam, Amsterdam, The Netherlands; 2 Microcirculation, Amsterdam Cardiovascular Sciences, Amsterdam, The Netherlands; 3 Atherosclerosis & Ischemic Syndromes, Amsterdam Cardiovascular Sciences, Amsterdam, The Netherlands; 4 Department of Surgery, Amsterdam University Medical Centers, Vrije Universiteit, Amsterdam, The Netherlands

**Keywords:** abdominal aortic aneurysm, mortality, cardiovascular disease, cardiovascular mortality

## Abstract

**Background:**

Patients with abdominal aortic aneurysms (AAAs) have poor survival rates after aneurysm repair compared with the general population, potentially due to increased cardiovascular risk. This systematic review and meta-analysis aimed to assess the long-term incidence of all-cause and cardiovascular mortality after elective, infrarenal AAA repair.

**Method:**

The Preferred Reporting Items for Systematic Review and Meta-Analysis (PRISMA) guidelines were followed (PROSPERO ID: CRD42022344547). Studies published in PubMed, Web of Science, and COCHRANE databases between January 2013 and May 2023 with a mean follow-up time of ≥5 years were included. A weighted linear regression analysis was performed to determine the annual incidence of all-cause and cardiovascular mortality five years after AAA repair. A random effects model calculated the overall incidence rates per 1000 person-years (PY). Endovascular aneurysm repair (EVAR) and open surgical repair (OSR) were compared.

**Results:**

Nineteen studies with 84 212 patients (mean follow-up: 68.9 [±13.3] months) were included. Common preoperative cardiovascular comorbidities included hypertension (74.4%), dyslipidemia (43.6%), and coronary artery disease (27.6%). At five years, the mean all-cause mortality was 29.78%, and cardiovascular mortality was 11.98%, with an annual increase of 6.59% and 2.46%, respectively (R^2^=0.809, p<0.001 and R^2^=0.824, p<0.001). The random effects model showed an all-cause mortality rate of 62.99 events (95% CI=57.53–68.96; *I*^2^=93%) per 1000 PY and a cardiovascular mortality rate of 24.19 events per 1000 PY (95% CI=21.69–26.98; *I*^2^=66%). Patients undergoing an EVAR had a significant higher incidence of all-cause and cardiovascular mortality than patients undergoing an OSR (B-coefficient 4.10 and 2.39, both p<0.001, respectively).

**Conclusion:**

The long-term all-cause and cardiovascular mortality remain high following elective, infrarenal AAA repair. These findings highlight a much needed optimization and emphasis of cardiovascular risk management, to minimize the long-term incidence of cardiovascular mortality in patients with AAA following surgical intervention.

**Clinical Impact:**

This study evaluated the long-term outcomes of cardiovascular and all-cause mortality rates following elective repair of the infrarenal abdominal aortic aneurysm. The results of this systematic review and meta-analysis emphasizes the suboptimal cardiovascular risk profile observed in this patient population. Futhermore, it highlights the importance of optimization and emphasis of cardiovascular risk management, including in the long-term after surgical intervention.

## Introduction and Rationale

Abdominal aortic aneurysms (AAAs) are a significant public health concern, with reported prevalence rates in the general population around 1%, predominantly affecting Caucasian male individuals.^1–3^ When compared with the general population, the life expectancy of patients with an AAA is significantly lower, and it is suggested that this is generally related to an increased cardiovascular risk.^4–9^

Current guidelines of the American Heart Association and National Institute for Health and Care Excellence recommend antiplatelet therapy, lipid-lowering agents, and antihypertensive drugs in patients with a high cardiovascular risk, to reduce the risk of cardiovascular events.^10^ Notably, previous studies have demonstrated improved 5-year survival rates for patients who adhere to these medication regimes.^9,11^ Specifically, AAA patients receiving statins (68.4% vs 42.2%), antiplatelet agents (63.6% vs 39.7%), or antihypertensive agents (61.5% vs 39.1%) exhibit elevated survival rates, compared with those not receiving these medications.

Notwithstanding the enhanced implementation of cardiovascular risk management among vascular patients, studies have demonstrated a persistent elevation in major adverse cardiovascular events (MACE) and death in AAA patients.^12^ Data from a systematic review and meta-analysis showed an elevated risk of cardiovascular mortality in patients with a small AAA, with an annual cardiovascular mortality rate of approximately 3% each year after the diagnosis, compared with an annual cardiovascular mortality rate in the general population in 1995 of 0.78%.^13^ Furthermore, a retrospective observational study found a persistent elevation in incidence rates of MACE and cardiovascular mortality in patients after endovascular aneurysm repair (EVAR), with an incidence of 22.4% and 11.6% at five years, respectively.^14^

While the risk of cardiovascular mortality has been extensively studied in the surveillance cohort and in the short term after AAA repair, there is a lack of studies focusing on the long-term risk of cardiovascular mortality in patients after AAA repair. Thus, this systematic review and meta-analysis aimed to assess and quantify the long-term incidence of cardiovascular and all-cause mortality rate after elective, infrarenal EVAR or open (OSR) AAA repair. Furthermore, the mortality rates of the treatment modalities were compared.

## Materials and Method

The Preferred Reporting Items for Systematic Review and Meta-Analysis (PRISMA) guidelines 2020 for systematic reviews and meta-analyses were followed for this study.^15^ The Grading of Recommendations, Assessment, Development, and Evaluation (GRADE) methodology was used to assess the certainty of evidence.

A scoping search was undertaken first to identify any relevant papers on this topic and concurrent studies on the same topic were checked in PROSPERO. This review was registered in PROSPERO (ID: CRD42022344547).

### Study Endpoints

The primary endpoint of the study was the all-cause and cardiovascular mortality rate at five years after elective, infrarenal AAA repair.

Furthermore, the annual incidence of all-cause and cardiovascular mortality thereafter was assessed. Cardiovascular mortality was defined as death resulting from cerebrovascular disease (CVD), AAA-related death, or death resulting from a MACE, encompassing acute coronary syndrome, de novo atrial fibrillation, heart failure, mitral valve insuffiency. and revascularization (including percutaneous coronary intervention (PCI) and coronary artery bypass graft surgery (CABG)).^12^

### Search

With the assistance of a clinical librarian, we performed a systematic literature search on PubMed, Web of Science, and COCHRANE to identify all English written studies from January 2013 until May 2023 (Supplemental Table S1). Studies were included if they reported on the rate of all-cause mortality or cardiovascular mortality of patients undergoing elective OSR or EVAR of infrarenal AAAs, with a mean or median follow-up of at least five years. Furthermore, studies not written in English, studies reporting on young patients (<18 years old), connective tissue disorder, and studies with a small sample size (less than 30 patients) were excluded. Finally, symptomatic, ruptured, and urgent AAAs, as well as thoracoabdominal, pararenal, juxtarenal, and isolated iliac aneurysms were excluded. Systematic literature reviews and meta-analysis captured in our database search were excluded as well, but used to cross check included studies and to identify any additional references.

### Study Selection

All studies identified with the search were sent to the review software Rayyan. Duplicates were removed, and two reviewers (S.E.M.v.K., C.F.) screened for title and abstract for inclusion/exclusion independently. Abstracts that did not report enough information were taken forward for full-text screening. Subsequently, a full-text review was performed and all inclusion/exclusion decisions were documented by the independent reviewers (S.E.M.v.K., C.F.). Any uncertainties were resolved by a third independent reviewer (K.K.Y.). A flow diagram was made to report on the screening process.

From the included studies, the following data were extracted by two reviewers (S.E.M.v.K., C.F.) into Microsoft Excel (Microsoft Corporation, Version 2016): study design, sample size, patient demographics (sex, age, comorbidities, medication), preoperative AAA diameter, AAA treatment (OSR or EVAR), mean or median follow-up of the cohort, study period, long-term all-cause mortality rate, and long-term incidence of cardiovascular mortality.

### Quality Assessment

The quality of randomized controlled trials (RCTs) was assessed using the ROBINS II tool.^16^ The quality of non-randomized cohort studies was assessed using the Newcastle-Ottawa Scale, whereas the quality of non-randomized non-comparative studies was assessed using the Joanna Briggs Institute Prevalence Critical Appraisal tool.^17,18^

Due to the nature of this study, analysis of publication bias was deemed irrelevant and was therefore not conducted.

### Statistical Analysis

All statistical analyses were conducted using SPSS (IBM SPSS Statistics, version 28) and R software (R Core Team, version 4.2.1, Vienna, Austria). A statistician was consulted for all statistical analysis to ensure accuracy (S.v.D.). Continuous variables were reported as mean value with a standard deviation (SD, ±), while categorical variables were expressed as frequencies. Median follow-up periods were converted into mean follow-up using the formula of Wan et al.^19^ N-weighted means were assessed for the preoperative patient characteristics and follow-up period. The Mann-Whitney *U* test was used to compare the preoperative patient characteristic between patients receiving EVAR and OSR.

The cardiovascular mortality and all-cause mortality rates were extracted and plotted against respective mean study follow-up period using n-weighted linear regression. The outcomes of the EVAR and OSR groups were compared using the B coefficient. Furthermore, a random effect model with an inverse variance method was used to pool the incidence years of all-cause and cardiovascular mortality after five years, expressed per 1000 person-years (PY). Heterogeneity was assessed by the *I*^2^ index, with an *I*^2^ >75% representing high heterogeneity. The results were plotted for the total cohort, and the EVAR and OSR groups separately.

When necessary, authors of studies were contacted to provide raw data for the analysis, maximally twice. If the authors did not answer within two weeks, the studies were excluded.

## Results

### Literature Search

**Figure 1.**
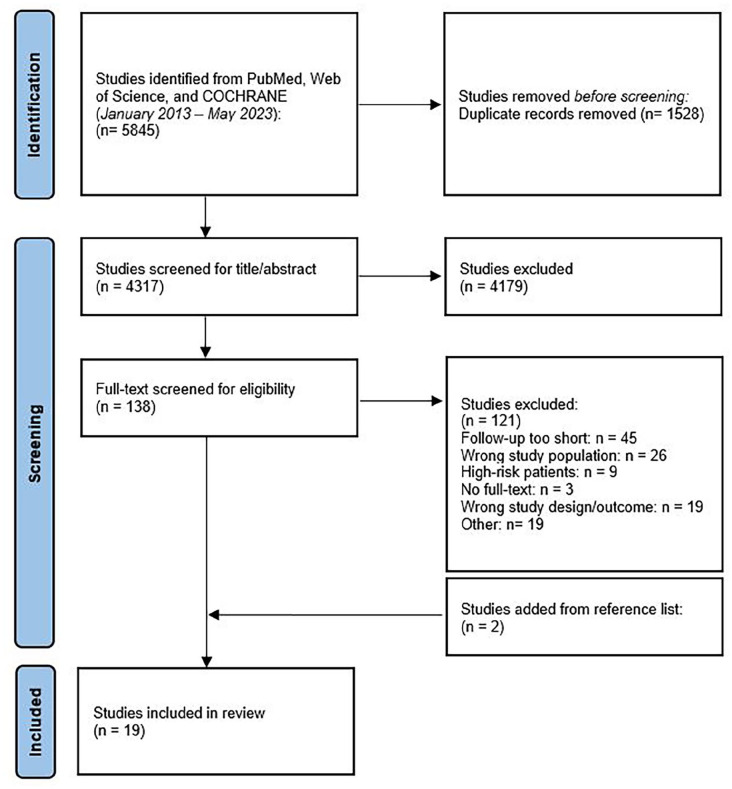
PRISMA diagram of selected studies.

A total of 5845 studies were identified from the search. Following removal of duplicates and excluding studies on title/abstract screening, 138 studies were taken forward for a full-text review. After full-text review and including two studies from the reference list, 19 were included for analysis.^20–38^ The results of the literature search are summarized in a flow diagram (Figure 1); the studies are reported in Supplemental Table S2.

The selected studies were as follows: three RCTs, ten non-randomized cohort studies, and six non-randomized non-comparative studies. Long-term all-cause mortality was reported in all 19 studies, whereas cardiovascular mortality was detailed in eight papers.^20,27,28,31,32,34,35,38^

The mean follow-up period of all patients was 68.9 (±13.3) months, with the longest follow-up being 152.4 months, the shortest 60 months.

From all 19 studies, 84 212 patients were included (82.6% male). For 4656 patients, rates of cardiovascular mortality were described.

The quality of the studies is shown in Supplemental Table S3; all studies scored moderate to high scores. The GRADE tool revealed low and very low evidence for the all-cause and cardiovascular mortality.

### Preoperative Cardiovascular Comorbidities and Medicine

Prevalence of the preoperative cardiovascular comorbidity profile in AAA patients was assessed. The most present comorbidity prior to AAA repair was hypertension (74.4%), followed by dyslipidemia (43.6%) and coronary artery disease (27.6%). The results are described in Table 1. The weighted means of the comorbidities in only RCTs and prospective studies are reported in Supplemental Table S4. One study had noticeable different prevalence of prescribed medicine.^33^ Therefore, weighted means without this study were also evaluated (Supplemental Table S5).

**Table 1.** Weighted Means—Preoperative Cardiovascular Comorbidities.

Comorbidity	Studies	Weighted mean (%)	Range (%)
Hypertension	^20,21,23,24,27–33,35–37^	74.4 (±6.6)	54.5–88.9
Heart failure	^27,33,34,37^	12.3 (2.8)	3.9–17.7
Diabetes mellitus	^20,21,23,24,27–38^	17.4 (±6.1)	7.4–30.8
Dyslipidemia	^20,21,23,27,29,30,32,34,36^	43.6 (±10.6)	29.5–64.7
Chronic renal failure	^21,23,24,27,29,30,32–34,37^	13.7 (6.9)	2.6–25.9
CAD	^21,23,24,27–30,32–34,36–38^	27.6 (±7.2)	16.9–53
PAD	^20,21,23,27,30,33,37^	9.4 (±5.4)	2.8–27.5
Cerebrovascular disease	^20,23,24,27,28,30,32–35^	11.8 (±5.4)	3.5–20.5
**Medication**			
Antiplatelet therapy	^21,28,33,34,36^	6.5 (±17.3)	0.1–63.4
Anticoagulants	^21,28,33,35,36^	8.3 (±2.9)	6.0–12.3
Lipid-modifying agents	^34,35^	49.5 (±15.3)	37–72.5
Beta-blocker	^28,33–35^	35.6 (±7.9)	33.7–87.2
ACE-inhibitor	^28,33,35^	48.2 (±4.5)	28–53.8

Values are presented in mean percentages (± SD) and the range in percentage.

Abbreviations: CAD: coronary artery disease; PAD: peripheral artery disease.

Furthermore, weighted means of preoperative comorbidities and medication between the EVAR and OSR groups were compared. All variables except the preoperative lipid-modifying agent use were significantly different between the two groups (p=0.242) (Table 2). Specifically, EVAR patients had a significantly higher prevalence of coronary artery disease (28.8% vs 21.8%), CVD (13.8% vs 4.8%), and chronic renal failure (15.9% vs 2.7%) compared to the OSR patients, and were also prescribed significantly more medication.

**Table 2.** Weighted Means—Preoperative Baseline Characteristics of EVAR and OSR Patients.

Comorbidity	EVAR weighted mean (N=69 280)	OSR weighted mean (N=14 693)	p (Mann-Whitney *U*-test)
Mean age, years	76.4 (±1.2)	71.4 (±0.7)	<0.001
AAA-diameter, mm	52 (±2.9)	59.9 (±2.8)	<0.001
**Hypertension**	73.9 (±6.9)	76.6 (±3.1)	<0.001
Heart failure	13.7 (±2.3)	9.7 (±1.7)	<0.001
Diabetes mellitus	16.5 (±6.0)	21.6 (±4.4)	<0.001
Dyslipidemia	42.2 (±11.1)	46.4 (±8.9)	<0.001
Chronic renal failure	15.9 (±5.1)	2.7 (±3.3)	<0.001
CAD	28.8 (±5.5)	21.8 (±10.5)	<0.001
PAD	6.9 (±4.0)	14.7 (±3.8)	<0.001
Cerebrovascular disease	13.8 (±4.1)	4.8 (±3.5)	<0.001
Medication			
Antiplatelet therapy	10.2 (±20.5)	4.1 (±14.6)	<0.001
Anticoagulants	11.7 (±1.6)	6.2 (±1.1)	<0.001
Lipid-modifying agents	53.7 (±17.7)	42	0.242
Beta blocker	39.3 (±9.8)	35.1 (±6.1)	<0.001
ACE-inhibitor	52.6 (±3.9)	45.8 (±2.3)	<0.001

Values are presented in mean percentages (± SD) unless indicated otherwise.

Abbreviations: CAD: coronary artery disease; PAD: peripheral artery disease.

### All-Cause Mortality and Cardiovascular Mortality

Data on all-cause mortality were provided in all included studies; ten studies described the all-cause mortality on patients with a history of EVAR,^20,22–24,30–32,34,36,37^ and one study on patients with a history of OSR.^27^ Eight studies provided data on the all-cause mortality on both EVAR and OSR patients.^21,25,26,28,29,33,35,38^ One of these studies did not make a differentiation between EVAR and OSR, and was therefore not included in the sub-analysis dividing both surgical treatment options.^26^

The mean age of all patients at the start of the study was 75.3 (±2.4) years. A total of 69 280 patients (82.3%) received endovascular AAA repair, whereas 14 693 patients (17.4%) had OSR. Preoperative mean AAA diameter was 52.5 mm (±3.5).

#### All-cause mortality

The long-term incidence of all-cause mortality after AAA repair was entered into an n-weighted linear regression model. In total, 29 219 all-cause deaths (34.7%) were recorded.

**Figure 2.**
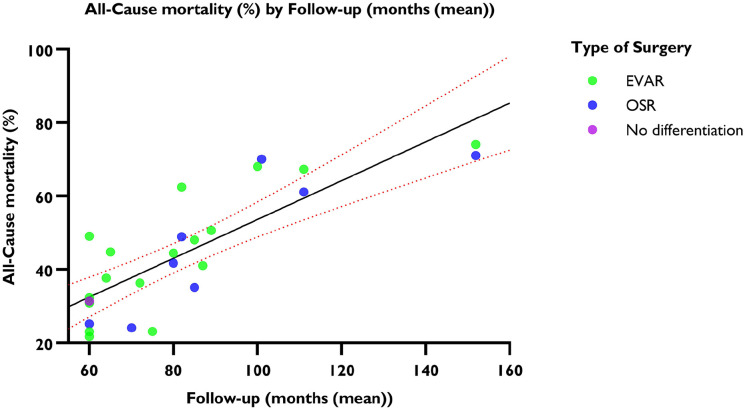
Weighted linear regression and all-cause mortality. The mean is shown with 95% CI. y-axis: all-cause mortality in percentages. x-axis: follow-up period in months.

**Figure 3.**
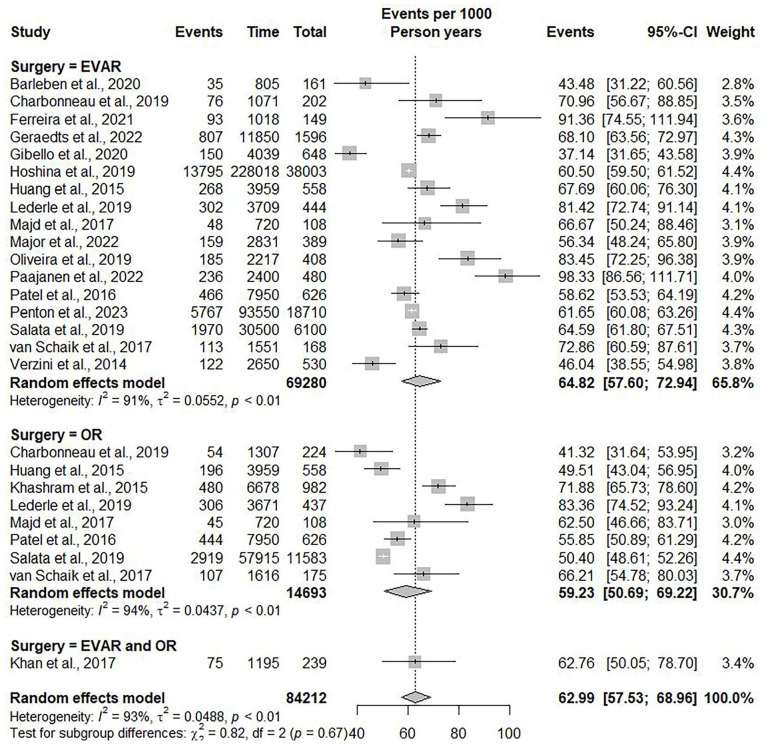
Forest plot showing the incidence rate of all-cause mortality per 1000 person-years with 95% confidence interval (CI), divided into EVAR and OSR.

The linear regression model suggested a positive correlation between follow-up duration and all-cause mortality (R^2^=0.809, p<0.001). Five years after AAA repair, the mean all-cause mortality was 29.78% (95% CI=29.46%–30.09%), with an annual increase of 6.59% (95% CI=6.56%–6.61%) (Figure 2). The random effects model revealed an overall incidence rate of all-cause mortality after five years of 62.99 events (95% CI=57.53–68.96; *I*^2^=93%) per 1000 PY (Figure 3).

In the subgroup of patients undergoing EVAR, 24 593 of the 69 280 (35.49%) patients died at a mean follow-up of 69.07 (±11.05) months. At 5-year follow-up, the all-cause mortality was 30.81% (95% CI=30.48%–31.08%), with an annual increase of 6.22% (95% CI=6.18%–6.24%) (R^2^(2)=0.757, p<0.001). The random effects model showed an incidence rate of all-cause mortality after five years in the EVAR group of 64.82 events (95% CI=57.60–72.94) per 1000 PY.

In the subgroup of patients undergoing OSR, 4451 of 14 693 (30.29%) patients died at a mean follow-up of 67.92 (±20.98) months. At 5-year follow-up, the all-cause mortality was 25.99% (95% CI=25.47%–26.51%), with an annual increase of 7.07% (95% CI=7.02%–7.12%) (R^2^=0.853, p<0.001). The random effects model showed an incidence rate of all-cause mortality after five years in the OSR group of 59.23 events (95% CI=50.69–69.22) per 1000 PY.

Patients undergoing an EVAR had a significant higher incidence of all-cause mortality than patients undergoing an OSR (B coefficient=4.10, p<0.001).

The all-cause mortality was frequently related to the cardiovascular events or malignancies. The incidence of malignancy-related mortality was reported between 1.8% and 20.1%.^20,21,28,31,32,34,35,38^ None of the studies that compared EVAR and OSR found a statistical significant difference of malignancy-related mortality.^21,28,35,38^ However, the majority of the studies indicated a high incidence of death from unidentified causes, ranging from 0.5% to 56.1%.^6,20,22,27,31,32,35,38^

#### Cardiovascular mortality

Overall, eight studies described the rates of long-term cardiovascular mortality or provided data via e-mail.^20,27,28,31,32,34,35,38^ Four studies included only EVAR patients,^20,31,32,34^ one study included OSR patients,^27^ and three studies included both EVAR and OSR patients.^28,35,38^ The mean follow-up was 101.9 (±33.8) months; the shortest mean follow-up was 60 months and the longest 152.4 months. In total, 4656 patients were included, and 957 patients (20.6%) died of cardiovascular causes in the follow-up period.

**Figure 4.**
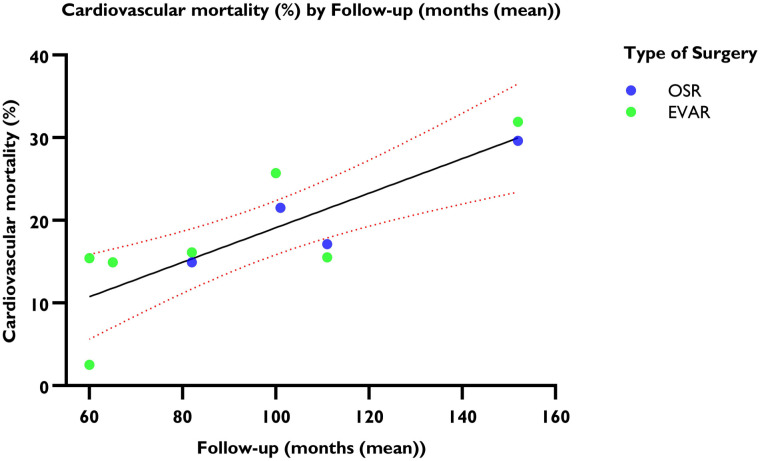
Weighted linear regression and cardiovascular mortality. The mean is shown with 95% CI. y-axis: cardiovascular mortality in percentages. x-axis: follow-up period in months.

**Figure 5.**
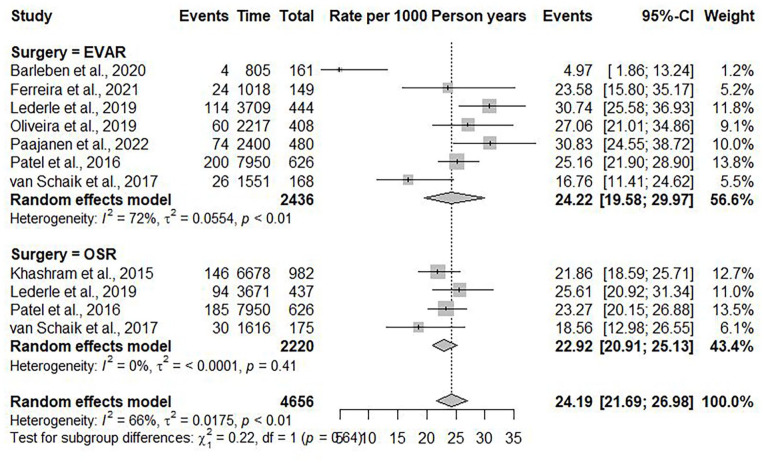
Forest plot showing the incidence rate of cardiovascular mortality per 1000 person-years with 95% confidence interval (CI), divided into EVAR and OSR.

The linear regression analysis suggested that 11.98% (95% CI=11.38%–12.58%) of the patients died of cardiovascular causes at five years, and the risk of cardiovascular mortality increased annually by 2.46% (95% CI=2.42%–2.50%) (R^2^=0.824, p<0.001) (Figure 4). The random effects model estimated an overall incidence rate of cardiovascular mortality of 24.19 events per 1000 PY (95% CI=21.69–26.98; *I*^2^=66%) (Figure 5).

In the subgroup of patients undergoing EVAR, 502 of the 2436 (20.6%) patients died of cardiovascular causes at a mean follow-up of 93.6 (±36.6) months. At five years, the cardiovascular mortality was 13.11% (95% CI=12.41%–13.87%), with an annual increase of 2.46% (95% CI=2.41%–2.52%) (R^2^=0.771, p<0.001). The random effects model estimated an incidence rate of cardiovascular mortality after five years in the EVAR group of 24.22 events per 1000 PY (95% CI=19.58–29.97).

In the subgroup of patients undergoing OSR, 455 of 2220 (20.5%) patients died of cardiovascular causes at a mean follow-up of 107.8 (±29.3) months. At five years, the cardiovascular mortality was 10.75% (95% CI=10.38%–11.12%), with an annual increase of 2.46% (95% CI=2.44%–2.48%) (R^2^=0.936, p<0.001). The random effects model estimated an incidence rate of cardiovascular mortality after five years in the OSR group of 22.92 events per 1000 PY (95% CI=20.91–25.13).

Patients undergoing an EVAR had a significant higher incidence of cardiovascular mortality than patients undergoing an OSR (B coefficient=2.39, p<0.001).

## Discussion

This systematic review and meta-analysis investigated the long-term all-cause and cardiovascular mortality in patients after elective, infrarenal AAA repair. An all-cause mortality rate of 29.78% at five years was found, with an increase of 6.59% yearly thereafter. The cardiovascular mortality rate reached 11.98% at the 5-year mark, with an annual increase of 2.46%. The random effects model found a mean incidence rate of 62.99 (95% CI=57.53–68.96) per 1000 PY for all-cause mortality and 24.19 (95% CI=21.69–26.98) per 1000 PY for the cardiovascular mortality. Similar results were found by two studies focusing on patients with unrepaired AAAs. Bath et al^13^ found an annual risk of cardiovascular mortality of 3%, and a recent meta-analysis by Sharma et al^39^ showed the mean incidence of cardiovascular death of 2.31 per 100 PY. In contrast, the annual cardiovascular death rate in the general population is estimated around 0.21%, as reported by the Office for National Statistics for England and Wales.^40^

While an impaired aneurysm-related mortality rate in patients undergoing EVAR compared with OSR is well known from data of large trials, this is the first study investigating the differences in long-term cardiovascular mortality rates.^41^ In our meta-analysis, we found results suggesting a higher long-term cardiovascular mortality rate in the EVAR group. It should be noted that this study is dealing with a selection bias, with patients subjected to EVAR exhibiting a more comprised cardiac profile compared with those undergoing an OSR. This is also reflected by the significantly higher percentages of medication prescription in the EVAR group and the preferred choice of treatment modality by the vascular surgeon. Open surgical repair appears to be the preferred choice for patients with more favorable cardiovascular risk profiles, as indicated in the lower incidence of cardiovascular mortality in the long term. Although the unavailability of raw patient data made a regression analysis of the effect of preoperative comorbidities infeasible, statistically significant differences in preoperative patient characteristics could therefore have influenced the differences in mortality rates. Nonetheless, both in the EVAR and OSR groups, the cardiovascular mortality surpasses that of the general population and may indicate inadequate cardiovascular risk management, especially those receiving an EVAR.

According to European Society of Vascular Surgery recommendations, patients with AAAs should receive basic lifestyle counseling and seek appropriate medical management for cardiovascular comorbidities.^10^ Studies have suggested prescription of cardiovascular medicines, and an increase in survival has been seen in patients receiving those medications.^9,37^ Despite this, our study does not demonstrate a clear downtrend in all-cause or cardiovascular mortality rates, regardless of the publication or inclusion dates of the analyzed studies.

The inconsistent outcome could be a result of the low rates of prescriptions and adherence of cardiovascular drugs.^42^ Data of Saratzis et al^11^ showed that only 63.2% of the patients with small AAA were prescribed both an antiplatelet agent and a statin, and not one of the included AAA screening units in England and Wales monitored compliance with the recommendations. Similar with their results, our results show a prescription rate of only 53.7% for antiplatelet therapy and 49.5% for lipid-modifying agents, irrespective of the exclusion of one study with disproportional low prescriptions.^33^ This diminished prescription rate could be attributed to the recent changes in the guidelines, recommending these medications. The introduction of cardiovascular risk management has been a gradual process, but the concept gained prominence only around 2000.^43,44^ Furthermore, the first World Health Organization (WHO) CVD risk chart was only published in 2007.^45^ The mean inclusion year of the studies included in our meta-analysis was 2008, with studies including patients dating back to 1997. Therefore, it is plausible that sufficient cardiovascular risk management was not yet applied.

Moreover, patients might not receive sufficient cardiovascular follow-up. Diender et al^12^ demonstrated a reduced incidence rate of MACE in patients with infrarenal AAA and pre-existing atrial fibrillation, 1 year following elective endovascular AAA repair, compared with patients without pre-existing atrial fibrillation. The reduction in MACE persisted after five years in patients with a history of valvular dysfunction, compared with those without. These findings accentuate a possible pivotal role of early cardiological follow-up in optimizing the challenge of current cardiovascular risk management and care of patients with AAAs. By facilitating timely surveillance, it enables clinicians to implement appropriate and targeted secondary prevention strategies and potentially reduce the cardiovascular mortality in the long term.^8,46^

A limitation of the study is the large heterogeneity in the all-cause mortality incidence rates. The retrospective nature of most studies and the wide variance of preoperative patient characteristics could have contributed to this wide range. Another limitation is the high percentage of unidentified deaths. Since many included studies are of an observational, retrospective design, causes of death are generally not described and non-traceable. It is likely that the rate of cardiovascular causes is underestimated in our study, and the large heterogeneity of the results is caused by the large proportion of unknown causes of death. Given the pathophysiological nature of AAA disease, it is expected that the likelihood of cardiovascular death is even higher in these patients.^47^ Furthermore, this systematic review focused on the patients who underwent elective, infrarenal AAA repair. Therefore, the results could be underestimated compared with patients undergoing urgent AAA repair, or patients with more complex AAA, like suprarenal, paravisceral, or thoracoabdominal aortic aneurysms.^48–51^

## Conclusions

This systematic review and meta-analysis found a high risk of cardiovascular mortality in the long term after elective, infrarenal AAA repair. Moreover, patients undergoing an EVAR showed a higher incidence in all-cause and cardiovascular mortality in the long term, compared with patients undergoing OSR. Our findings show that OSR patients generally have a more favorable preoperative risk profile, which may suggest that OSR patients undergo more thorough preoperative screening than those receiving EVAR. However, despite these advantages, both groups exhibit worse survival rates compared with the general population. These results highlight a much needed optimization of cardiovascular risk management in AAA patients, while under surveillance after aortic surgery, to reduce the long-term incidence of cardiovascular mortality.^5^

## Supplemental Material

sj-docx-1-jet-10.1177_15266028241304627 – Supplemental material for Long-term Cardiovascular and All-Cause Mortality following Elective Infrarenal Repair of the Abdominal Aortic Aneurysm: A Systematic Review and Meta-analysis Supplemental material, sj-docx-1-jet-10.1177_15266028241304627 for Long-term Cardiovascular and All-Cause Mortality following Elective Infrarenal Repair of the Abdominal Aortic Aneurysm: A Systematic Review and Meta-analysis by Samira E. M. van Knippenberg, Cecilia Fenelli, Susan van Dieren, Ronak Delewi, Ron Balm and Kak Khee Yeung in Journal of Endovascular Therapy
