# Supplementary material for: Long-term Cardiovascular and All-Cause Mortality following Elective Infrarenal Repair of the Abdominal Aortic Aneurysm: A Systematic Review and Meta-analysis
Source: J Endovasc Ther. 2024 Dec 30;33(3):1115–25. doi: 10.1177/15266028241304627 (PMC13172124; doi:10.1177/15266028241304627)
Supplement: sj-docx-1-jet-10.1177_15266028241304627 – Supplemental material for Long-term Cardiovascular and All-Cause Mortality following Elective Infrarenal Repair of the Abdominal Aortic Aneurysm: A Systematic Review and Meta-analysis [file sj-docx-1-jet-10.1177_15266028241304627.docx]

Longterm Cardiovascular and All-cause Mortality following Elective Infrarenal Repair of the Abdominal Aortic Aneurysm: A Systematic Review and Meta-analysis

**Supplementary Table 1**: Literature search

| **PubMed** | ((abdominal AND aortic AND (aneurysm* OR aneurism*)) OR (AAA))  AND  (heart failure OR acute coronary syndrome OR myocardial infarction OR heart attack OR angina, stable OR angina OR angina pectoris OR angina unstable OR coronary disease OR myocardial ischemia OR myocardial ischaemia OR ischaemic heart disease OR ischaemic attack, transient OR TIA OR stroke OR brain infarct OR cerebral infarction OR brain infarction OR cardiovascular mortality)  AND  ("2013/01/01"[Date - Publication] : "2023/05/04"[Date - Publication]) |
| --- | --- |
| **Cochrane Library** | Cochrane Database of Systematic Reviews  Cochrane Central Register of Controlled Trials  ID Search Hits  #1 (((abdominal AND aortic AND (aneurysm* OR aneurism*)) OR (AAA))):ti,ab,kw  #2 ((heart failure OR acute coronary syndrome OR myocardial infarction OR heart attack OR angina, stable OR angina OR angina pectoris OR angina unstable OR coronary disease OR myocardial ischemia OR myocardial ischaemia OR ischaemic heart disease OR ischaemic attack, transient OR TIA OR stroke OR brain infarct OR cerebral infarction OR brain infarction OR cardiovascular mortality)):ti,ab,kw  #3 #1 and #2 with Cochrane Library publication date Between Jan 2013 and May 2023 |
| **Web of Science** | ((abdominal AND aortic AND (aneurysm* OR aneurism*)) OR (AAA)) (All Fields) and (heart failure OR acute coronary syndrome OR myocardial infarction OR heart attack OR angina, stable OR angina OR angina pectoris OR angina unstable OR coronary disease OR myocardial ischemia OR myocardial ischaemia OR ischaemic heart disease OR ischaemic attack, transient OR TIA OR stroke OR brain infarct OR cerebral infarction OR brain infarction OR cardiovascular mortality) (All Fields) and 2023 and 2022 or 2021 or 2020 or 2019 or 2018 or 2017 or 2016 or 2015 or 2014 or 2013(Publication Years) |

**Supplementary Table 2**: Description of the included articles

| **Study** | **Year** | **Surgery type** | **Followup (mean, months)** | **Total AAA-patients** | **Mean age (years)** | **Male (%)** | **Mean AAA-diameter (mm)** | **Diabetes Mellitus (%)** | **Hypertension (%)** | **Dyslipidemia (%)** | **Chronic Renal Failure (%)** | **CAD (%)** | **Myocardial infarction (%)** | **PAD (%)** | **Heart Failure (%)** | **Cerebrovascular Disease (%)** | **Antiplatelet therapy(%)** | **Anticoagulans (%)** | **Lipid modifying agents (%)** | **Beta blocker (%)** | **ACE-inhibitor (%)** | **Diabetic medication (%)** | **All-cause mortality (%)** | **Cardiovascular-related Death (%)** |
| --- | --- | --- | --- | --- | --- | --- | --- | --- | --- | --- | --- | --- | --- | --- | --- | --- | --- | --- | --- | --- | --- | --- | --- | --- |
| ^1^ | 2019 | EVAR | 60 | 161 | 73 | 87.6 |  | 34 (21.1) | 136 (84.5) | 113 (70.2) | 22 (13.7) |  |  | 38 (23.6) |  | 21 (13) |  |  |  |  |  |  | 35 (21.7) | 4 (2.5) |
| ^2^ | 2019 | EVAR | 64 | 202 | 78.1 | 78.7 | 58.3 | (22.3) | (69.3) | (56.4) | (11.9) | (46) |  | (12.4) |  |  | (56.9) | (11.9) |  |  |  |  | 76 (37.6) |  |
|  |  | OR | 70 | 224 | 70.9 | 80.45 | 56.7 | (17.4) | (79) | (64.7) | (17) | (45.1) |  | (18.3) |  |  | (60.7) | (8.5) |  |  |  |  | 54 (24.1) |  |
| ^3^ | 2021 | EVAR | 82 | 149 | 72.6 | 88 | 59.6 | 24 (16.1) |  | 44 (29.5) | 24 (16.1) | 66 (44.3) |  |  | 13 (8.7) | 17 (11.4) | 83 (55.7) |  | 108 (72.5) | 130 (87.2) |  |  | 93 (62.4) | 24 (16.1) |
| ^4^ | 2022 | EVAR | 89 | 1596 | 73.5 | 89 | 61 |  |  |  |  |  |  |  |  |  |  |  |  |  |  |  | 807 (50.6) |  |
| ^5^ | 2020 | EVAR | 75 | 648 | 74.8 | 93.5 | 55.6 | 139 (21.4) | 441 (68.1) | 205 (31.6) | 109 (16.8) | 337 (52) |  | 175 (27) |  | 133 (20.5) |  |  |  |  |  |  | 150 (23.1) |  |
| ^6^ | 2017 | EVAR | 72 | 38003 | 77 | 82.7 | 51 | 4611 (12.1) | 26124 (68.7) |  | 7333 (19.3) | 10713 (28.2) |  |  |  | 5861 (15.4) |  |  |  |  |  |  | 13795 (36.3) |  |
| ^7^ | 2015 | OR | 85 | 558 | 72 | 86 | 59 |  |  |  |  |  |  |  |  |  |  |  |  |  |  |  | 196 (35.1) |  |
|  |  | EVAR | 85 | 558 | 74 | 86 | 57 |  |  |  |  |  |  |  |  |  |  |  |  |  |  |  | 268 (48) |  |
| ^8^ | 2018 | EVAR | 60 | 239^a^ |  |  |  |  |  |  |  |  |  |  |  |  |  |  |  |  |  |  | 75 (31.4) |  |
|  | 2018 | OR |  |  |  |  |  |  |  |  |  |  |  |  |  |  |  |  |  |  |  |  |  |  |
| ^9^ | 2016 | OR | 82 | 982 | 72.1 | 78.4 | 59.3 | 107 (10.9) | 703 (71.6) | 399 (40.6) | 80 (8.1) | 438 (44.6) |  | 270 (27.5) | 38 (3.9) | 129 (13.1) |  |  |  |  |  |  | 480 (48.9) | 146 (14.9) |
| ^10^ | 2019 | EVAR | 100 | 444 |  | 99.3 | 57 | 100 (22.5) | 347 (78.2) |  |  | 174 (39.2) | 105 (23.6) |  |  | 67 (15.1) | 244 (55) | 44 (9.9) |  | 282 (63.5) | 192 (43.2) |  | 302 (68) | 114 (25.7) |
|  |  | OR | 101 | 437 |  | 99.5 | 57 | 100 (22.9) | 330 (75.5) |  |  | 185 (42.3) | 110 (25.2) |  |  | 70 (16) | 277 (63.4) | 34 (7.8) |  | 282 (64.5) | 180 (41.2) |  | 306 (70) | 94 (21.5) |
| ^11^ | 2017 | EVAR | 80 | 108 | 71.8 | 98.1 | 59.3 | 10 (9.3) | 96 (88.9) | 62 (57.4) | 28 (25.9) | 56 (51.9) |  |  |  |  |  |  |  |  |  |  | 48 (44.4) |  |
|  |  | OR | 80 | 108 | 71.9 | 98.1 | 60.2 | 8 (7.4) | 96 (88.9) | 55 (50.9) | 28 (25.9) | 56 (51.9) |  |  |  |  |  |  |  |  |  |  | 45 (41.7) |  |
| ^12^ | 2019 | EVAR | 87 | 389 | 74.1 | 82 | 54 | 54 (13.9) | 214 (55) | 142 (36.5) | 18 (4.6) | 124 (31.9) |  | 47 (12.1) |  | 26 (6.7) |  |  |  |  |  |  | 160 (41) |  |
| ^13^ | 2019 | EVAR | 65 | 408 | 72.6 | 87.9 |  | 68 (11) | 281 (69.6) |  |  |  |  |  |  |  |  |  |  |  |  |  | 185 (45.8) | 60 (14.9) |
| ^14^ | 2022 | EVAR | 60 | 480 | 76 | 87 | 62 | 96 (20) | 337 (70) | 239 (50) | 99 (21) | 252 (53) |  |  |  | 59 (12) |  |  |  |  |  |  | 236 (49) | 74 (15.4) |
| ^15^ | 2016 | EVAR | 152 | 626 | 74.1 | 90 | 64 | 61 (10) |  |  |  | 269 (43) |  |  |  |  |  |  |  |  |  |  | 466(74) | 200 (31.9) |
|  |  | OR | 152 | 626 | 74 | 91 | 65 | 68 (11) |  |  |  | 261 (42) |  |  |  |  |  |  |  |  |  |  | 444 (71) | 185 (29.6) |
| ^16^ | 2023 | EVAR | 60 | 18710 |  | 81.2 |  | (20.8) | (82.8) |  | (13.6) | (30.3) |  | (7.2) | (12.4) |  |  |  |  |  |  |  | 5767 (30.8) |  |
| ^17^ | 2019 | EVAR | 60 | 6100 | 75.3 | 84.6 |  | 1877 (30.8) | 5052 (82.4) |  | 161 (2.6) | 1082 (17.7) | 373 (6.1) | 169 (2.8) | 1082 (17.7) | 78 (3.7) | 6 (0.1) | 750 (12.3) |  | 2208 (36.2) | 3279 (53.8) | 774 (12.7) | 1970 (32.3) |  |
|  |  | OR | 60 | 11583 | 71.2 | 78.8 |  | 2711 (23.4) | 8944 (77.2) |  | 197 (1.7) | 1957 (16.9) | 640 (5.5) | 1567 (13.5) | 1183 (10.2) | 191 (3.5) | 31 (0.3) | 696 (6) |  | 3903 (33.7) | 5355 (46.2) | 1012 (8.7) | 2919 (25.2) |  |
| ^18^ | 2017 | OR | 111 | 175 | 69.6 | 90 |  | (9.6) | (54.5) | (52.6) | (8.4) | (46.6) |  |  |  | (15.2) | 70 (41) | 27 (15) | 72 (42) | 92 (52) | 50 (28) |  | 107 (61.1) | 30 (17.1) |
|  |  | EVAR | 111 | 168 | 70.7 | 93 |  | (10.4) | (58.4) | (47) | (7.5) | (41) |  |  |  | (14.5) | 72 (40) | 20 (12) | 63 (37) | 76 (44) | 58 (34) |  | 113 (67.3) | 26 (15.5) |
| ^19^ | 2014 | EVAR | 60 | 530 | 72.2 | 91.5 | 53.2 | 53 (10) | 370 (69.8) | 197 (37.2) |  | 558 (48.7) |  |  |  |  | 256 (48.3) | 35 (6.4) |  |  |  |  | 122 (23) |  |

**Supplementary Table 2**: Description of the included articles and pre-operative characteristics; Pre-operative characteristics are presented in N (%); CAD = coronary artery disease, PAD = peripheral artery disease. ^a^Includes both EVAR and OSR-patients.

**Supplementary Table 3.** Risk of Bias and GRADE assessment

| **Supplementary Table JBI Table Quality Assessment: non-comparative studies/Prevalence studies** | | | | | | | | | | | | | | | | | | | | | | | |
| --- | --- | --- | --- | --- | --- | --- | --- | --- | --- | --- | --- | --- | --- | --- | --- | --- | --- | --- | --- | --- | --- | --- | --- |
| **Author** | | **Year** | | | **Sample frame** | | **Sampling** | | **Adequate sample size** | | **Setting and study subjects** | **Data analysis with sufficient coverage** | | **Identification of condition** | | **Measurement of condition** | | **Appropriate statistical analsyis** | | **Response rate** | | **Total score (Maximum of 9 points)** | |
| **Kharshram M. (11)** | | 2016 | | | 1 | | 0 | | 1 | | 1 | 1 | | 1 | | 1 | | 1 | | 1 | | 8 | |
| **Barleben A. (1)** | | 2019 | | | 1 | | 1 | | 1 | | 1 | 1 | | 0 | | 0 | | 1 | | 0/1 | | 6/7 | |
| **Khan N. (10)** | | 2017 | | | 1 | | 0 | | 1 | | 1 | 1 | | 1 | | 0/1 | | 1 | | 1 | | 7/8 | |
| **Hoshina K. (8)** | | 2019 | | | 1 | | 1 | | 1 | | 1 | 1 | | 1 | | 0 | | 1 | | 1 | | 8 | |
| **Oliveira N. F.G. (15)** | | 2019 | | | 1 | | 1 | | 1 | | 1 | 1 | | 1 | | 1 | | 1 | | 1 | | 9 | |
| **Penton A. (19)** | | 2023 | | | 1 | | 1 | | 1 | | 0 | 1 | | 0 | | 0 | | 0 | | 1 | | 5 | |
| **Supplementary Table. NOS for the risk of bias and quality assessment of NRSs** | | | | | | | | | | | | | | | | | | | | | | | |
| **Author** | **Year** | | **Selection** | | | | | | | | | | | | **Comparability** | | **Exposure** | | | | | | **Total score (Maximum of 9 points)** |
|  |  | | | **Retrospective/Case control** | | **Adequate definition of patient cases/representativeness of the exposed cohort** | | **Representativeness of patient cases/selection of the non-exposed cohort** | | **Selection of controls/Ascertainment of exposure** | | | **Definition of controls/Outcome of interest** | | **Control for important or additional factors** | | **Ascertainment of exposure/Assessment of outcome** | | **Same method of ascertainment for participants/Follow-up** | | **Nonresponse rate/Adequacy of follow-up** | |  |
| **Ferreira R. S. (3)** | 2021 | | | Retrospective study of prospective collected database | | 1 | | 1 | | 1 | | | 1 | | 2 | | 1 | | 1 | | 0 | | 8 |
| **Geraedts A.C.M. (6)** | 2022 | | | Retrospective | | 1 | | 1 | | 1 | | | 1 | | 1 | | 1 | | 1 | | 1 | | 8 |
| **Gibello L.. (7)** | 2020 | | | Retrospective study of prospective collected database | | 1 | | 1 | | 1 | | | 1 | | 2 | | 1 | | 1 | | 0 | | 8 |
| **Paajanen P. (17)** | 2022 | | | Retrospective | | 1 | | 1 | | 1 | | | 0 | | 2 | | 1 | | 1 | | 0 | | 7 |
| **Charbonneau P. (2)** | 2019 | | | Retrospective | | 1 | | 1 | | 1 | | | 1 | | 1 | | 0 (partially through EPD, partially through interviews) | | 1 | | 1 | | 7 |
| **Major M. (14)** | 2019 | | | Retrospective | | 1 | | 1 | | 1 | | | 1 | | 0 | | 1 | | 1 | | 1 | | 7 |
| **Majd P. (13)** | 2017 | | | Retrospective study of prospective collected database | | 1 | | 1 | | 1 | | | 1 | | 2 | | 1 | | 1 | | 1 | | 9 |
| **Salata K. (20)** | 2019 | | | Retrospective | | 1 | | 1 | | 1 | | | 0 | | 2 | | 1 | | 1 | | 1 | | 8 |
| **Verzini F. (22)** | 2014 | | | Retrospective study of prospective collected database | | 1 | | 1 | | 1 | | | 1 | | 0 | | 1 | | 1 | | 1 | | 7 |
| **Huang Y. (9)** | 2015 | | | Retrospective | | 1 | | 1 | | 1 | | | 1 | | 2 | | 1 | | 1 | | 1 | | 9 |

| **Unique ID** | **Study ID** | **Experimental** | **Comparator** | **Outcome** | **Weight** | **D1** | **D2** | **D3** | **D4** | **D5** | **Overall** |  |  |  |
| --- | --- | --- | --- | --- | --- | --- | --- | --- | --- | --- | --- | --- | --- | --- |
| **Lerdere F.A. (12)** | 1 | Endovascular Repair | Open Repair | Long-term Mortality | 1 |  |  |  |  |  |  |  |  | Low risk |
| **van Schaik T.G. (21)** | 2 | Endovascular Repair | Open Repair | Long-term Mortality | 1 |  |  |  |  |  |  |  |  | Some concerns |
| **Patel R. (18)** | 3 | Endovascular Repair | Open Repair | Long-term Mortality | 1 |  |  |  |  |  |  |  |  | High risk |

**Supplementary Table 3. Risk of Bias –** Randomized Controlled Trials using the ROBINS-II tool
D1. Randomnisation process; D2. Deviations from the intended interventions; D3. Missing outcome data; D4. Measurement of the outcome; D5. Selection of the reported result.; quality of non-randomized cohort studies was assessed using the Newcastle-Ottawa Scale; quality of non-randomized non-comparative studies was assessed using the Joanna Briggs Institute Prevalence Critical Appraisal-tool.

**Supplementary Table 4**. Weighted means pre-operative comorbidities and medication: Randomized Controlled Trials and Prospective studies

| **Comorbitidy** | **Studies** | **Weighted mean** | **Range** |
| --- | --- | --- | --- |
| **Male** | ^1, 9, 10, 15, 18^ | 89,3 (±7,6) | 78,4 – 99,5 |
| **Hypertension** | ^1, 9, 10, 18^ | 72,2 (±7,5) | 54,5 – 84,5 |
| **Heart failure** | ^9^ | 3,9 | - |
| **Diabetes Mellitus** | ^1, 9, 10, 15, 18^ | 14,0 (±5,4) | 9,6 – 22,9 |
| **Dyslipidemia** | ^1, 9, 18^ | 45,9 (±9,4) | 40,6 – 70,2 |
| **Chronic renal failure** | ^1, 9, 18^ | 8,6 (1,8) | 7,5 – 13,7 |
| **Coronary Artery Disease (CAD)** | ^9, 10, 15, 18^ | 42,8 (±1,9) | 39,2 – 46,6 |
| **Peripheral artery Disease (PAD)** | ^1, 9^ | 26,9 (1,4) | 23,6 – 27,5 |
| **Cerebrovascular disease** | ^1, 9, 10, 18^ | 14,2 (±1,9) | 13,0 - 16 |
| **Medication** | **Studies** | **Weighted mean** | **Range** |
| **Antiplatelet therapy** | ^10, 18^ | 53,9 (±9,1) | 40 – 63,4 |
| **Anticoagulants** | ^10, 18^ | 10,2 (±2,4) | 7,8 - 15 |
| **Lipid modifying agent** | ^18^ | 39,6 (±2,5) | 37 – 42 |
| **Beta blocker** | ^10, 18^ | 59,5 (±7,5) | 28 – 43,2 |
| **ACE-inhibitor** | ^10, 18^ | 39,1 (±5,4) | 28 – 43,2 |

Values are presented in mean percentages (±SD)

**Supplementary Table 5:** weighted means of pre-operative medication use

| **Medication** | **Studies** | **Weighted mean** | **Weighted mean, all studies excl. Salata et al. ^17^ :** | **Range** |
| --- | --- | --- | --- | --- |
| **Antiplatelet therapy** | ^2, 3, 10, 17, 19^ | 6,5 (±17,3) | 53,7 (±7,5) | 0,1 – 63,4 (without Salata et al. ^17^: 40 – 63,4) |
| **Anticoagulants** | ^2, 10, 17-19^ | 8,3 (±2,9) | 9,2 (±2,5) | 6 – 15 (without Salata et al.^17^: 6,4 – 15) |
| **Lipid-modifying agents** | ^3, 18^ | 49,5 (±15,3) | 49,5 (±15,3) | 37 – 72,5 |
| **Beta blocker** | ^3, 10, 17, 18^ | 35,6 (±7,9) | 62,5 (±11,1) | 33,7 – 87,2 (without Salata et al.^17^: 44 – 87,2) |
| **ACE-inhibitor** | ^10, 17, 18^ | 48,2 (±4,5) | 39,1 (±5,4) | 28 – 53,8 (without Salata et al.^17^: 28 – 43,2) |

Values are presented in mean percentages (±SD)

1. Barleben A, Mathlouthi A, Mehta M, Nolte T, Valdes F, Malas MB. Long-term outcomes of the Ovation Stent Graft System investigational device exemption trial for endovascular abdominal aortic aneurysm repair. J Vasc Surg. 2020;72(5):1667-73.e1.

2. Charbonneau P, Hongku K, Herman CR, Habib M, Girsowicz E, Doonan RJ, et al. Long-term survival after endovascular and open repair in patients with anatomy outside instructions for use criteria for endovascular aneurysm repair. J Vasc Surg. 2019;70(6):1823-30.

3. Soares Ferreira R, Oliveira-Pinto J, Ultee K, Voûte MT, Oliveira NFG, Hoeks S, et al. Long Term Outcomes of Post-Implantation Syndrome After Endovascular Aneurysm Repair. Eur J Vasc Endovasc Surg. 2021;62(4):561-8.

4. Geraedts ACM, Mulay S, Vahl AC, Verhagen HJM, Wisselink W, de Mik SML, et al. Editor's Choice - Post-operative Surveillance and Long Term Outcome after Endovascular Aortic Aneurysm Repair in Patients with an Initial Post-operative Computed Tomography Angiogram Without Abnormalities: the Multicentre Retrospective ODYSSEUS Study. Eur J Vasc Endovasc Surg. 2022;63(3):390-9.

5. Gibello L, Varetto G, Ruffino MA, Peretti T, Frola E, Cieri E, et al. Long Term Outcomes of Endovascular Aortic Repair in Patients With Abdominal Aortic Aneurysm and Ectatic Common Iliac Arteries. Eur J Vasc Endovasc Surg. 2020;60(3):356-64.

6. Hoshina K, Ishimaru S, Sasabuchi Y, Yasunaga H, Komori K. Outcomes of Endovascular Repair for Abdominal Aortic Aneurysms: A Nationwide Survey in Japan. Ann Surg. 2019;269(3):564-73.

7. Huang Y, Gloviczki P, Oderich GS, Duncan AA, Kalra M, Fleming MD, et al. Outcome after open and endovascular repairs of abdominal aortic aneurysms in matched cohorts using propensity score modeling. J Vasc Surg. 2015;62(2):304-11.e2.

8. Khan N, Lyytikäinen LP, Khan J, Seppälä I, Lehtomäki A, Kuorilehto T, et al. Extended Serum Lipid Profile Predicting Long-Term Survival in Patients Treated for Abdominal Aortic Aneurysms. World J Surg. 2018;42(4):1200-7.

9. Khashram M, Jenkins JS, Jenkins J, Kruger AJ, Boyne NS, Foster WJ, Walker PJ. Long-term outcomes and factors influencing late survival following elective abdominal aortic aneurysm repair: A 24-year experience. Vascular. 2016;24(2):115-25.

10. Lederle FA, Kyriakides TC, Stroupe KT, Freischlag JA, Padberg FT, Jr., Matsumura JS, et al. Open versus Endovascular Repair of Abdominal Aortic Aneurysm. N Engl J Med. 2019;380(22):2126-35.

11. Majd P, Ahmad W, Becker I, Brunkwall JS. Ten-Year Single-Center Results of Abdominal Aortic Aneurysm Treatment: Endovascular versus Open Repair. Ann Vasc Surg. 2017;44:113-8.

12. Major M, Long GW, Eden CL, Studzinski DM, Callahan RE, Brown OW. Long-term outcomes and interventions of postoperative type 1a endoleak following elective endovascular aortic aneurysm repair. J Vasc Surg. 2022;75(1):136-43.e1.

13. Oliveira NFG, Ultee K, van Rijn MJ, Pinto JP, Raa ST, Bastos Gonçalves F, et al. Anatomic predictors for late mortality after standard endovascular aneurysm repair. J Vasc Surg. 2019;69(5):1444-51.

14. Paajanen P, Lindström I, Oksala N, Väärämäki S, Saari P, Mäkinen K, Kärkkäinen JM. Radiographically quantified sarcopenia and traditional cardiovascular risk assessment in predicting long-term mortality after endovascular aortic repair. J Vasc Surg. 2022;76(4):908-15.e2.

15. Patel R, Sweeting MJ, Powell JT, Greenhalgh RM. Endovascular versus open repair of abdominal aortic aneurysm in 15-years' follow-up of the UK endovascular aneurysm repair trial 1 (EVAR trial 1): a randomised controlled trial. Lancet. 2016;388(10058):2366-74.

16. Penton A, DeJong M, Zielke T, Nam J, Blecha M. The Impact of Perioperative Morbidities, Lack of Discharge Aspirin, and Lack of Discharge Statin on Long Term Survival Following EVAR. Vasc Endovascular Surg. 2023:15385744231173198.

17. Salata K, Hussain MA, de Mestral C, Greco E, Aljabri BA, Mamdani M, et al. Comparison of Outcomes in Elective Endovascular Aortic Repair vs Open Surgical Repair of Abdominal Aortic Aneurysms. JAMA Netw Open. 2019;2(7):e196578.

18. van Schaik TG, Yeung KK, Verhagen HJ, de Bruin JL, van Sambeek M, Balm R, et al. Long-term survival and secondary procedures after open or endovascular repair of abdominal aortic aneurysms. J Vasc Surg. 2017;66(5):1379-89.

19. Verzini F, Isernia G, De Rango P, Simonte G, Parlani G, Loschi D, Cao P. Abdominal aortic endografting beyond the trials: a 15-year single-center experience comparing newer to older generation stent-grafts. J Endovasc Ther. 2014;21(3):439-47.

**References**
